# Interprofessional global health competencies of South Korean health professional students: educational needs and strategies

**DOI:** 10.1186/s12909-019-1826-1

**Published:** 2019-11-21

**Authors:** Junghee Kim, Hyeonkyeong Lee, In Sook Kim, Tae Wha Lee, Gwang Suk Kim, Eunhee Cho, Kyung Hee Lee

**Affiliations:** Mo-Im Kim Nursing Research institute, College of Nursing, Yonsei University, Seoul, 50-1 Yonsei-ro, Seodaemun-gu, Seoul, 03722 South Korea

**Keywords:** Competency, Educational needs, Global health, Health professional students, Interprofessional education, Sustainable development goals

## Abstract

**Background:**

There is growing recognition of the importance of educating health professional students to enhance their competence in collaborating with individuals from other health professions in the area of global health. This study aimed to identify the performance levels in interprofessional global health competencies (IGHC) of health professional students, their educational needs, and the strategies for successfully developing IGHC.

**Methods:**

This study used a mixed methods design involving an online survey followed by focus group interviews. A sample of 325 fourth-year undergraduate students from 14 health-related majors completed a self-report online survey (38.8% response rate). The performance of IGHC was measured on a five-point Likert scale using the IGHC items developed by the Consortium of Universities for Global Health. Additionally, 12 senior students and five professors in global health-related majors participated in focus group interviews. The students’ educational needs and priorities were analysed using the Borich needs assessment and the Locus for Focus model.

**Results:**

The participants’ IGHC mean score was 3.11 (SD = 0.55) and differed by previous global health activity experiences (t = − 2.10, *p* = .037). Nine competencies in six domains using the Locus for Focus model were identified as a priority for global health education. Suggested strategies to enhance IGHC included establishing IGHC education in formal curricula, developing value-based content and outcomes, and engaging students in learning activities.

**Conclusions:**

It is necessary to design an interprofessional pre-departure course to achieve the priority IGHC and to organise learning activities where there is cooperation in problem solving while applying the expertise of each major within resource-limited settings. This study supports future health professional education that should foster enhanced roles and scopes of practice as changing agents to assure the achievement of sustainable development goals.

## Background

There is growing recognition of the importance of educating health professional students to enhance their competence as global health professionals. Addressing transnational health problems requires collaboration among health professionals in diverse fields [1, 2]. Accordingly, academic programs preparing health professionals are becoming more aware of the importance of interprofessional education (IPE) in preparing future global health practitioners. IPE involves the simultaneous, dialogic, and collaborative education of students from different disciplines, ultimately improving health outcomes for their patients [3, 4].

Research findings have provided support for the importance of IPE for health professional students. In one study [5] students who had participated in IPE were found to have positive attitudes toward team-based activities. Team-based interventions, in turn, have a positive impact on patients’ health index and boost health professionals’ work performance [6]. Through their participation in IPE programs designed for students from more than six health-related disciplines, students recognised the value of working in IPE teams and had improved communication skills with vulnerable populations, such as refugees and immigrants [1]. While the evidence on the positive influence of IPE on the development of communication skills was inconclusive, a review reported a similar finding, indicating nursing and medical students’ positive attitude change toward interprofessional collaboration through IPE programs [7]. IPE seems to be invaluable in the development of health professionals who are able to respond to global health issues in a collaborative way [4]. However, there is lack of evidence on the impact of IPE programming on global health.

In 2015, members of the Global Health Competency Subcommittee of the Consortium of Universities for Global Health (CUGH) proposed four levels of interprofessional global health competencies (IGHC): (1) global citizen, (2) exploratory, (3) basic operational, and (4) advanced [8]. The first level refers to competencies that students in every field should gain; the second, to competencies required for students who are pursuing "global health or preparing for a global health field experience"; the third, to competencies needed by students in health professions who will likely devote part of their professional work to global health issues; and the fourth, to competencies that are "more discipline-specific or tailored to the job or capacity in which one is working" [8]. The subcommittee members proposed 8 domains for the first level and 39 competencies in 11 domains for the third. They acknowledged the need for additional research and discussion to validate the proposed levels and competencies.

This study aimed to (1) describe and compare by participants’ characteristics their performance levels in IGHC as proposed by the CUGH subcommittee; (2) identify their educational needs based on their perceptions of the importance and their performance level in IGHC; and (3) explore strategies for enhancing IGHC education in South Korea.

## Methods

### Design

This study used a mixed methods design that included quantitative and qualitative components. The former involved an online survey questionnaire sent to health professional students at a university in South Korea to identify their perceptions of the importance of and their performance in the 39 IGHC identified by the CUGH subcommittee. The latter comprised focus group interviews with students and faculty members concerning IPE and strategies for improving it in global health work.

### Sample and setting

The target population for this study was 837 fourth-year students (the entrance quota as of 2014) at a large university in South Korea who were enrolled in one of the 14 health-related majors (i.e., nursing, medicine, dentistry, pharmacy, social welfare, food and nutrition, environmental engineering, medical engineering, public health, clinical pathology, physical therapy, occupational therapy, radiology, and dental hygiene). As response rates for online surveys tend to be less than 21% [9–11], we targeted the entire population of 837 in the current study to reach the required sample size. Further details are described elsewhere [12]. Although 526 (62.8%) students agreed to participate, only 374 responded to the survey (44.7% of the target population). Forty-nine questionnaires with missing data were excluded from the analysis, resulting in a final sample of 325 (38.8% of the target population).

Two focus group interviews were conducted with 12 students, divided into two groups with six members each. The participants were seniors majoring in health professional disciplines who had participated in activities related to global health during their undergraduate program through student clubs, volunteer work, or academic curriculum, and who understood the research objective and agreed to participate in the study. The participants were recruited through a purposive sampling method through department offices, global health-related student clubs, and coordinators of global health programs. The students who participated in the focus group interviews were all nursing students. Eleven of the twelve students in the focus groups were female, and one was male. An additional focus group interview was carried out with professors teaching global health subjects for college or graduate students in global health majors, who had experience in conducting short-term global health training programs. The participants in the expert group were two nursing professors and three medical professors.

### Instruments

Perceived performance and importance of IGHC were measured using the domains and competencies in the basic operational program-oriented levels (Level III) [8]. It was translated into Korean using the committee approach to translation [13, 14]. Two bilingual committee members with doctorates in nursing, as well as research experience in global health or IPE, independently translated the instrument into Korean. The principal investigator and two translators examined the validity of the IGHC translation and agreed on the contents. The measure comprises 39 competency items in 11 domains, adding 16 items to the original 13 items in eight domains of the global citizen level and adding 10 items in three domains (Domain 4: capacity strengthening; Domain 9: program management; and Domain 11: strategic analysis) of the basic operational program-oriented level. Respondents were asked to rate the importance of these competencies and their perceived level of performance in each competency with respect to access to educational need on a 5-point Likert scale (1 = “very low”, 5 = “very high”).

Open-ended questions were used in the focus group interviews. The questions in the student focus group interviews were as follows: 1) “What is your experience of participating in the global health program in college?” 2) “How about the experience of interacting with other department students in college?” 3) “What is your class experience with other department students in college?” 4) “What is needed to increase the global health learning experience among healthcare students?” Each question included the positive and negative aspects of experience.

The expert group interview was conducted based on the results of the analysis of the students’ educational needs. The questions in the expert group interview were as follows: 1) “What is the most important thing in IGHC?” 2) “What is the priority ability to be developed in the process of education?” 3) “What is most lacking in the current curriculum?” 4) “What aspect of competence needs to be developed most intensively in the domestic situation?” 5) “What do you think of strategies for improving IGHC?”

### Data collection

Data for the online survey were collected for approximately 10 weeks, from April 25 to June 30, 2017. To ensure coverage of the population, the researchers asked the administrators of student online communities for cooperation by posting recruitment notices of the online survey system’s URL (http://ko.surveymonkey.com) on their websites. Additionally, all 14 administrative offices were notified through an official letter with a recruitment notice. Following this, the researchers provided the recruitment notice online and offline by using the online board or social networking service (SNS) of those accepted departments.

The focus group interviews were based on the focus group interview guidelines suggested by Krueger and Casey [15]. The interviews lasted between 60 and 90 min and were conducted in a seminar room in the university where the interviewees felt comfortable. Using training materials, graduate students who were trained and experienced in qualitative research explained the process, which included the major questions to be asked, took notes of the participants’ non-verbal actions and facial expressions, and tape-recorded all the conversations during the interview. The expert group interview was conducted with five global health experts in the form of a group discussion, facilitated by the principal investigator.

### Ethical considerations

The study protocol was reviewed and approved by the Institutional Review Board of on May 23, 2017 (IRB No. 2017–0004). Through the online survey introduction, the objective and procedures of the study were explained, and participants were informed of the risks and benefits of participation, voluntary nature of their participation, the possibility of withdrawing from the study whenever the participant wanted to, and the anonymity and confidentiality of the data collected. Those who agreed to participate in the online survey were asked to click “I agree” before proceeding to the research questions. Before the focus group interview, the participants were informed of the objective and method of the research, as well as data anonymity and their right to withdraw from the interview. Written informed consent was obtained from each focus group participant.

### Data analysis

Descriptive statistics regarding frequency, mean, standard deviation, numbers, and percentages were collected using SPSS Win 20.0. A t-test and analysis of variance (ANOVA) were conducted for comparing the participants’ perceived level of performance in IGHC by general characteristics. Educational needs of the participants based on their IGHC score were identified using the equation proposed by Borich [16]. In addition, the educational needs were visualised using the Locus for Focus model, which was used as the base data for deciding relative priority [17]. The qualitative data were analysed using content analysis. After the student focus group interviews, the facilitator transcribed all the data; each data transcription took about 7 h. Two researchers (JH and HK) analysed the transcripts independently and met to reach agreement on the content. They reviewed the transcripts multiple times to identify meaningful words, sentences, and phrases, and to differentiate and categorise the data. They extracted the themes independently for each code, held further discussion, and then selected the theme. The expert discussions were analysed in the same way as the student focus group interviews. After two researchers (JH and HK) conducted independent analysis, they met and agreed on the process.

## Results

### Participants’ characteristics

The participants’ mean age was 23.5 years (SD = 2.35). The response rate was highest among nursing students (23.4%), followed by medical (14.2%), biomedical engineering (12.9%), and dental hygiene (10.8%) students. Dentistry (0.9%) and environmental engineering (0.6%) had the lowest response rates. Most (52.6%) did not have any experience living abroad. A small proportion of the students had been abroad for less than 6 months (26.2%), had engaged in exchange programs with foreigners or student clubs (19.7%) and took global health-related classes (20.3%). They were engaged in global health-related seminars, student clubs, and overseas activities (9.8%), and had experienced student clubs in and out of the school (26.8%). However, more than half of the participants (56.9%) took classes with students from other departments.

### IGHC level and its difference by general characteristics

The participants’ average performance score in IGHC was 3.11 (SD = 0.55) out of 5.00 (Table 1). Their mean scores in two out of 11 domains, namely, Domain 3 (social and environmental determinants of health; M = 3.34, SD = 0.78) and Domain 7 (professional practice; M = 3.34, SD = 0.70), were the highest, and that of Domain 1 (global burden of Disease; M = 2.79, SD = 0.72) was the lowest. The item score for “Describe the relationship between access to and quality of water, sanitation, food, and air on individual and population health” in Domain 3 was the highest (M = 3.73, SD = 0.90). The item score for “Describe major public health efforts to reduce disparities in global health (such as Millennium Development Goals and Global Fund to Fight AIDS, TB, and Malaria)” in Domain 1 was the lowest (M = 2.50, SD = 0.96).

**Table 1:** Participants’ perceived level of interprofessional global health competencies (*N* = 325)

Item		M ± SD
DOMAIN 1. Global Burden of Disease		
1. Describe the major causes of morbidity and mortality around the world, and how the risk for disease varies across regions.		2.70 ± 0.86
2. Describe major public health efforts to reduce disparities in global health (such as Millennium Development Goals and Global Fund to Fight AIDS, TB, and Malaria).		2.50 ± 0.96
3. Validate the health status of populations using available data (e.g., public health surveillance data, vital statistics, registries, surveys, electronic health records, and health plan claims data).		3.18 ± 0.98
Subtotal		2.79 ± 0.72
DOMAIN 2. Globalization of Health and Health care		
4. Describe different national models or health systems for provision of health care and their respective effects on health and health care expenditure.		2.71 ± 1.00
5. Describe how global trends in health care practice, commerce and culture, multinational agreements, and multinational organizations contribute to the quality and availability of health and health care locally and internationally.		2.66 ± 0.97
6. Describe how travel and trade contribute to the spread of communicable and chronic diseases.		3.19 ± 1.02
7. Describe general trends and influences in the global availability and movement of health care workers.		2.69 ± 1.01
Subtotal		2.82 ± 0.77
DOMAIN 3. Social and Environmental Determinants of Health		
8. Describe how cultural context influences perceptions of health and disease.		3.23 ± 0.94
9. List major social and economic determinants of health and their effects on the access to and quality of health services and on differences in morbidity and mortality between and within countries.		3.07 ± 1.00
10. Describe the relationship between access to and quality of water, sanitation, food, and air on individual and population health.		3.73 ± 0.90
Subtotal		3.34 ± 0.78
DOMAIN 4. Capacity Strengthening		
11. Collaborate with a host or partner organization to assess the organization’s operational capacity.		3.07 ± 0.95
12. Cocreate strategies with the community to strengthen community capabilities, and contribute to reduction in health disparities and improvement community health.		3.14 ± 0.92
13. Integrate community assets and resources to improve the health of individuals and populations.		2.94 ± 1.00
14. Identify methods for assuring program sustainability (proposed by members of the CUGH Global Health Competency Subcommittee).		2.89 ± 0.94
Subtotal		3.01 ± 0.75
DOMAIN 5. Collaboration, Partnering, and Communication		
15. Include representatives of diverse constituencies in community partnerships and foster interactive learning with these partners.		3.01 ± 0.89
16. Demonstrate diplomacy and build trust with community partners.		3.18 ± 0.93
17. Communicate joint lessons learned to community partners and global constituencies.		3.01 ± 0.98
18. Exhibit interprofessional values and communication skills that demonstrate respect for, and awareness of, the unique cultures, values, roles/responsibilities, and expertise represented by other professionals and groups that work in global health.		3.13 ± 0.90
19. Acknowledge one’s limitations in skills, knowledge, and abilities.		3.55 ± 0.95
20. Apply leadership practices that support collaborative practice and team effectiveness.		3.34 ± 0.88
Subtotal		3.20 ± 0.65
DOMAIN 6. Ethics		
21. Demonstrate an understanding of and an ability to resolve common ethical issues and challenges that arise when working within diverse economic, political, and cultural contexts as well as when working with vulnerable populations and in low-resource settings to address global health issues.		3.16 ± 0.87
22. Demonstrate an awareness of local and national codes of ethics relevant to one’s working environment.		3.11 ± 0.95
23. Apply the fundamental principles of international standards for the protection of human subjects in diverse cultural settings.		3.18 ± 0.93
Subtotal		3.15 ± 0.76
DOMAIN 7. Professional Practice		
24. Demonstrate integrity, regard, and respect for others in all aspects of professional practice.		3.66 ± 0.94
25. Articulate barriers to health and health care in low-resource settings locally and internationally.		3.21 ± 0.86
26. Demonstrate the ability to adapt clinical or discipline-specific skills and practice in a resource-constrained settings.		3.16 ± 0.89
Subtotal		3.34 ± 0.70
DOMAIN 8. Health Equity and Social Justice		
27. Apply social justice and human rights principles in addressing global health problems.		3.14 ± 0.95
28. Implement strategies to engage marginalized and vulnerable populations in making decisions that affect their health and well-being.		3.03 ± 0.96
29. Demonstrate a basic understanding of the relationships between health, human rights, and global inequities.		3.20 ± 0.92
30. Describe role of WHO in linking health and human rights, the Universal Declaration of Human Rights, International Ethical Guidelines for Biomedical Research Involving Human Subjects.		2.95 ± 1.00
31. Demonstrate a commitment to social responsibility.		3.49 ± 0.94
32. Develop understanding and awareness of the health care workforce crisis in the developing world, the factors that contribute to this, and strategies to address this problem.		3.00 ± 0.95
Subtotal		3.13 ± 0.69
DOMAIN 9. Program Management		
33. Plan, implement, and evaluate an evidence-based program.		3.26 ± 1.02
34. Apply project management techniques throughout program planning, implementation, and evaluation.		3.13 ± 0.95
Subtotal		3.20 ± 0.89
DOMAIN 10. Sociocultural and Political Awareness		
35. Describe the roles and relationships of the major entities influencing global health and development.		3.02 ± 0.96
Subtotal		3.02 ± 0.96
DOMAIN 11. Strategic Analysis		
36. Identify how demographic and other major factors can influence patterns of morbidity, mortality, and disability in a defined population.		3.26 ± 0.93
37. Conduct a community health needs assessment.		3.15 ± 0.89
38. Conduct a situation analysis across a range of cultural, economic, and health contexts.		3.20 ± 0.89
39. Design context-specific health interventions based on situation analysis.		3.14 ± 0.94
Subtotal		3.19 ± 0.69
	Total	3.11 ± 0.55

The perceived performance score in IGHC of those who had experienced global health-related seminars, student clubs, or overseas activities (M = 3.31, SD = 0.61) was significantly higher compared to the score of those without such experience (M = 3.09, SD = 0.54; t = − 2.10, *p* = .037; Table 2). There was no significant difference in IGHC by department (F = 1.18, *p* = .307) nor was there any significant difference in IGHC by any other general characteristic.

**Table 2:** Interprofessional global health competencies by participants’ general characteristics (*N* = 325)

Characteristics	Categories	N (%)	Interprofessional Global Health Competencies		
			M ± SD	t/F	*p*
Gender	Male	137 (42.2)	3.16 ± 0.56	1.25	.214
	Female	188 (57.8)	3.08 ± 0.54		
Department	Nursing	76 (23.4)	3.10 ± 0.52	1.18	.307
	Medicine	46 (14.2)	3.18 ± 0.60		
	Biomedical Engineering	42 (12.9)	3.12 ± 0.53		
	Dental Hygiene	35 (10.8)	2.93 ± 0.51		
	Radiological Science	30 (9.2)	2.97 ± 0.48		
	Health Administration	26 (8.0)	3.17 ± 0.47		
	Social Welfare	18 (5.5)	3.27 ± 0.65		
	Food and Nutrition	16 (4.9)	3.19 ± 0.40		
	Biomedical Laboratory Science	10 (3.1)	3.07 ± 0.44		
	Others^a^	26 (8.0)	3.26 ± 0.76		
Living overseas (month)	0	171 (52.6)	3.04 ± 0.54	2.32	.076
	< 6	85 (26.2)	3.16 ± 0.52		
	6–< 12	32 (9.8)	3.19 ± 0.61		
	≥12	37 (11.4)	3.26 ± 0.59		
Experiences of exchange with foreigners	No	261 (80.3)	3.09 ± 0.54	−1.74	.082
	Yes	64 (19.7)	3.22 ± 0.57		
Experiences of exchange with healthcare professional discipline students	No	229 (70.5)	3.08 ± 0.54	−1.56	.120
	Yes	96 (29.5)	3.19 ± 0.56		
Participation in global health classes	No	259 (79.7)	3.08 ± 0.54	−1.86	.064
	Yes	66 (20.3)	3.22 ± 0.57		
Global health activities	No	293 (90.2)	3.09 ± 0.54	−2.10	.037
	Yes	32 (9.8)	3.31 ± 0.61		
Participation in college union club	No	238 (73.2)	3.09 ± 0.56	−0.97	.335
	Yes	87 (26.8)	3.16 ± 0.53		
Class experiences with other major students	No	140 (43.1)	3.10 ± 0.60	−0.36	.721
	Yes	185 (56.9)	3.12 ± 0.51		

^a^ Dentistry (*n* = 3), environmental engineering (*n* = 2), occupational therapy (*n* = 8), pharmacy (n = 8), physical therapy (*n* = 5)

### Educational needs and IGHC

The top 19 priority competencies which were an average score of 2.23 or greater in the weighted discrepancy score were identified by applying the Borich needs assessment model (Table 3). Two competencies with the highest discrepancy score between importance and performance of IGHC, indicating the highest educational needs, were in Domain 1: “Describe the major causes of morbidity and mortality around the world and how the risk for disease varies with regions” and “Describe major public health efforts to reduce disparities in global health.” The other priority competencies were included in six domains: Domain 2 (globalization of health and health care), Domain 3 (social and environmental determinants of health), Domain 4 (capacity strengthening), Domain 5 (collaboration, partnering, and communication), Domain 7 (professional practice), and Domain 8 (health equity and social justice).

**Table 3:** Needs assessment of interprofessional global health competencies (*N* = 325)

Domain	Item	Discrepancy Score	Importance Level (Mean)	Weighted Discrepancy Score	Weighted Discrepancy Score Rank	Quadrant 1 (Locus for Focus Model)
1	1	0.94	3.64	3.44	1	
1	2	0.90	3.41	3.07	2	
8	32	0.79	3.79	3.01	3	v
4	13	0.79	3.72	2.93	4	v
8	28	0.75	3.79	2.86	5	v
2	5	0.80	3.47	2.78	6	
4	14	0.73	3.62	2.65	7	
4	11	0.69	3.77	2.61	8	v
4	12	0.66	3.80	2.50	9	v
5	15	0.67	3.68	2.46	10	
7	26	0.64	3.80	2.43	11	v
5	16	0.64	3.81	2.43	12	v
2	6	0.63	3.83	2.43	13	v
2	4	0.71	3.42	2.42	14	
3	9	0.64	3.71	2.39	15	
6	21	0.63	3.79	2.38	16	v
5	17	0.64	3.65	2.34	17	
2	7	0.68	3.37	2.28	18	
11	39	0.60	3.73	2.23	19	
1	3	0.57	3.75	2.12	20	
5	18	0.57	3.70	2.12	21	
7	25	0.56	3.77	2.11	22	
9	34	0.56	3.69	2.08	23	
6	22	0.56	3.67	2.07	24	
8	30	0.58	3.53	2.07	25	
11	37	0.55	3.71	2.05	26	
6	23	0.55	3.73	2.04	27	
8	29	0.54	3.74	2.00	28	
8	27	0.54	3.68	1.99	29	
3	8	0.53	3.76	1.99	30	
11	38	0.51	3.71	1.90	31	
9	33	0.50	3.76	1.86	32	
11	36	0.49	3.75	1.82	33	
10	35	0.50	3.53	1.77	34	
8	31	0.44	3.92	1.71	35	
5	20	0.44	3.79	1.68	36	
3	10	0.39	4.12	1.61	37	
7	24	0.31	3.97	1.23	38	
5	19	0.26	3.81	0.97	39	

*Note.* Domain 1. Global Burden of Disease; Domain 2. Globalization of Health and Health care; Domain 3. Social and Environmental Determinants of Health; Domain 4. Capacity Strengthening; Domain 5. Collaboration, Partnering, and Communication; Domain 6. Ethics; Domain 7. Professional Practice; Domain 8. Health Equity and Social Justice; Domain 9. Program Management; Domain 10. Sociocultural and Political Awareness; Domain 11. Strategic Analysis

Applying the Locus for Focus model, nine priority competencies in the first quadrant had a large difference in importance and performance (Table 3). Priority competencies were included in six domains: Domain 2 (globalization of health and health care), Domain 4 (capacity strengthening), Domain 5 (collaboration, partnering, and communication), Domain 6 (ethics), Domain 7 (professional practice), and Domain 8 (health equity and social justice). Most of these nine competencies were added in the basic operational program-oriented level, except for two (Nos. 6 and 21). According to Bloom’s taxonomy of educational objectives, two competencies were classified as knowledge (Nos. 6 and 32), three as knowledge and skill (Nos. 12, 13, and 28), one as attitude (No. 16), one as skill (No. 11), one as attitude and skill (No. 26) and one as knowledge, attitude, and skill (No. 21).

### Students’ suggestions to enhance IGHC

#### Respect for diversity and the shared value of interprofessional collaboration

The students in the focus group mentioned that it is important for students from various majors to have an open mind, accept different views, recognise their own limits, respect other majors, and cooperate with each other to enhance their learning experience in global health. In addition, they stated that it is necessary to help them realise that they can have more creative activities and outcomes, by not focusing only on sharing duties but understanding that they have common goals and taking on various views throughout their continuous education.

#### Motivation and self-directed

The participants stated that at the start of recruiting students into a program, the primary tasks must be to clarify the ultimate goal and meaning of the program, motivate them, and share more details of the schedule and program. It is necessary to enable students to take part in planning the program. They also mentioned the importance of planning self-initiated programs and giving motivation.

### Experts’ perspectives on IGHC

#### Needs of interprofessional cooperation in the formal educational curriculum

Under the current educational curriculum, learning experiences with students from other departments are insufficient and limited to only extracurricular activities, such as voluntary work in student clubs. The expert group agreed to include interprofessional cooperation in the formal educational curriculum as it serves as an important factor in solving issues in clinical practice and in the field of global health, as well as in affecting the medical outcome of patients. Therefore, within the entire educational curriculum, key competencies must be connected and continued throughout the school years and among different subjects.

#### Value-based core contents and outcomes of IGHC education

The experts argued that the following must be considered when deciding on the content to be included: the necessity of raising awareness of health equality (a determinant of social health); considering the role of social culture and politics, and engagement in local community; having an open mindset, sympathy, care, and respect for interprofessional roles; having communication, partnership, and respect for others; and enhancing the capacity to put into practice better health equality in resource-limited settings. In addition, they all agreed that humanities and sociology must be part of the curriculum, in addition to healthcare-related and clinical knowledge.

#### Engaging students in learning activities

Theoretical, practical, and professional competences must be developed. In terms of institutions, the experts mentioned that physical spaces where students of various majors can experience practical learning together must be provided; social voluntary work must be a requisite. They also suggested that virtual campus and qualitative materials (e.g. sharing best practices for indirect experience by using simulation and media), inviting students to study abroad, guest lectures with experts, education connecting history and politics in multicultural regions, and engagement of students from less developed countries would together be an effective teaching strategy for the latter or the disadvantaged to have more access to the site.

Experts and students in the focus group had the same opinion regarding the importance of interprofessional collaboration. However, the students said they needed to participate in the global health program from planning stage while the experts mainly addressed practical strategies (e.g. physical spaces, engagement of global students, participation of students from underdeveloped countries) for increasing student participation.

## Discussion

Although IPE has become an essential part of the recent healthcare curriculum [18, 19], there is no educational framework for interdisciplinary approaches to solving global health issues. The phasic structure of CUGH’s IGHC reflects the differing needs of healthcare professionals based on their level of engagement with global health practice after their graduation [8]. The current study focused on Level 3 of IGHC, which is necessary for health professionals to devote part of their professional work to global health and involved students from 14 healthcare majors to evaluate their educational needs. By doing so, we provided evidence for the refinement of Level 3 towards a more effective IGHC framework. In addition, our study is the first to contribute to the development of educational curricula that encourage IGHC among training health professionals in Korea.

Two competencies with the highest educational needs using the Borich needs assessment in Level 3 of IGHC were in the global burden of disease domain, which is required if students are to work in global health after graduation. This includes an understanding of the prevalence and mortality rates of diseases developing across the world; explaining major public health efforts to address the income gap among high-, middle-, and low-income countries, and reduce the gap in global health [8]. This begins at the global citizen level and should be enhanced further in the basic operational program-oriented level. The competency with the lowest perceived performance in this domain was “Describe major public health efforts to reduce disparities in global health”. Global health is defined as the discipline of collaborative transnational research and action for promoting health for all in the world, recognising that health is determined by problems, issues, and concerns that transcend national boundaries [20]. Therefore, a primary need of global health professionals is the competency to understand the burdens and causes of diseases faced by countries worldwide and to understand the gap between countries. It is also necessary to understand that global health issues cannot be solved solely by certain health professionals but instead cross the boundaries of different fields, including technology and bio-engineering, politics, and administration.

Moreover, it is important to recognise the importance of global efforts and policy directions needed to solve problems and to identify the role of each major in global healthcare. In colleges, students must acquire knowledge and technologies on sustainable development goals (SDGs), proposed in 2015. They must also be motivated and develop their competency through field practice [21]. As SDGs, which are designed to solve global issues and causes, require efforts to combine each discipline’s expertise, it is important to prepare students for IGHC. The fruits of such an approach, when thoughtfully implemented, are demonstrated in a collaboration between engineering and medicine students who developed diagnostic kits for high-risk pregnant women based on their learning activities in resource-limited and less developed countries [22].

Meanwhile, risk awareness in the health workforce and its causes, which were placed as the high priority using the Locus for Focus model in this study, are newly demanded knowledge at Level 3 of IGHC. Understanding the lack of a health workforce and its causes in resource-limited countries would not only expand the horizon of students in global health but also provide opportunities to search for strategies to solve workforce shortage issues in terms of health equality and social justice [23]. Moreover, because the participants in this study demonstrated a high need for learning how globalization affects the healthcare environment, it is necessary to create educational content that offers knowledge on the impact of globalization on the use and quality of healthcare both in and out of Korea. It is important to help them understand changes in healthcare environments caused by accelerated globalization in contexts, such as the negative impact of fast food companies that have emerged in less developed countries on the citizens’ dietary life, as well as the limits to accessing medical resources among immigrants and refugees [24]. In addition, the participants mentioned that education is needed to shift the perspectives of healthcare professionals so that they think of fulfilling their roles in South Korea as a part of globalization and global health [25].

In fact, students with overseas activities related to global health demonstrated a higher performance level in IGHC than those without such experiences, meaning that a well-established IPE in global health is required within the formal curriculum. As suggested by the experts in this study, the primary tasks are to clearly set and guide the expected outcomes through IPE programs in global health and to motivate students. For instance, an IPE program designed for students majoring in nursing and health in two universities in Hong Kong had set expected outcomes whereby students must realise the need of cooperation to solve problems; compare the roles, responsibilities, and limitations of other majors; and respect and listen to different views [26]. In the current study, as the most prioritised IGHC need for students was knowledge level competencies, such as explaining the global burden of disease and the influence of globalization on health, which need to precede overseas activities. As the first step to enhance IGHC, therefore, it is recommended to set a pre-departure course among different majors to educate students and set their expectations.

Another important factor for a successful global health program in less developed countries is the preparedness of students. The students in this study suggested that an open mindset focused on others’ views and behavioural patterns, embracing different views, recognising one’s own limits, having respect for other majors, and an attitude toward cooperation are important for a successful IPE program. This finding is consistent with the competencies outlined by UNESCO [27] that suggest teaching students to reach SDGs, which include self-awareness, collaboration, partnership competencies, and being comfortable in interdisciplinary settings. In addition, the following competencies were recommended to be included in pre-training programs: humility to focus carefully on the world’s context with regard to history, sociocultural system, and economy, and excellence in understanding continuity in different cultural contexts [28]. Social responsibility and an ethical awareness of the need to take care of people in resource-limited areas are suggested as being essential areas in which healthcare professionals must be prepared [29]. Such competencies must be built up through training in the global health field. However, they should also be emphasised when pre-departure education programs begin.

The growing trend toward more collaborative approaches in the healthcare environment is an essential strategy for promoting the quality of health services and improving the health outcomes of patients and communities, especially in resource-limited settings. In addition, IPE in global health may help to enhance future health professionals’ roles and their scopes of practice as changing agents to support the achievement of SDGs. This study provides adequate and timely evidence in support of training health professionals in a manner that will benefit the healthcare sector. Moving forward, it is important for educators and policy makers to integrate global health competencies into existing curricula to prepare students to become globally competent health professionals.

Since academic curricula and extracurricular activities can vary by department, it can be challenging to place students in interprofessional settings. Online learning could be an effective way to overcome such barriers. For effective communication, it is also important to design the physical space of collaboration in a way that does not reflect the traditional hierarchy of positions since collaboration can be difficult under such circumstances [4]. Professional culture, which refers to the ways of thinking and behaviour that differentiate one group of people from another, can pose a barrier to IPE [30]. Therefore, the presence of student and faculty leaders can help to alter negative attitudes about other professions and help to sustain interprofessional collaboration [4, 31]. In this study, the selection of participants did not take into account the proportion of students in each department. Although there was no difference in overall IGHC level by department, it is possible there were different characteristics related to IGHC within each discipline or department. Our intention, however, was to present evidence regarding the level of IGHC and strategies for IPE in global health among all health-related students. As it was not sufficient to include all possible needs and strategies for enhancing IGHC of students from different disciplines, further research is suggested needed to explore non-nursing students’ perspectives about the strategies for IPE.

## Conclusions

As the interdisciplinary approach to solve global health issues is gaining more importance, this study is significant because it is the first study that applies IGHC, published by CUGH, to graduating students from 14 healthcare majors. By doing so, this study identified the need for IGHC-building interventions to be developed as IPE programs in which students can work together to solve problems locally and globally. Therefore, designing teaching content and methods and adding the expertise of each major in resource-limited settings are important to enable the priority competencies of IGHC to become actual outcomes of a program. In addition, it is relevant to design pre-departure training programs so that students can understand the context of multicultural regions and be ready to engage in IPE.

## Data Availability

Not applicable.

## Abbreviations

CUGH: Consortium of Universities for Global Health
IGHC: Interprofessional Global Health Competencies
IPE: Interprofessional Education
SDGs: Sustainable Development Goals
SNS: Social Network Service
UNESCO: United Nations Educational, Scientific and Cultural Organization
